# Pregnancy Following Spontaneous Healing of Uterine Rupture: A Case Report and Experience of Management

**DOI:** 10.7759/cureus.70322

**Published:** 2024-09-27

**Authors:** Shunya Sugai, Kazufumi Haino, Kaoru Yamawaki, Kosuke Yoshihara, Koji Nishijima

**Affiliations:** 1 Obstetrics and Gynecology, Niigata University Medical and Dental Hospital, Niigata, JPN; 2 General Center for Perinatal, Maternal and Neonatal Medicine, Niigata University Medical and Dental Hospital, Niigata, JPN

**Keywords:** conservative treatment, maternal morbidity, recurrence, subsequent pregnancy, uterine rupture

## Abstract

Uterine rupture can heal naturally without the need for surgical intervention. However, reports on subsequent pregnancies are limited. A 27-year-old woman, gravida 2, para 1, visited our institution at seven weeks of gestation. She had previously experienced uterine rupture with postpartum hemorrhage, which had healed naturally without surgical intervention. We thoroughly explained the perinatal complications associated with the subsequent pregnancy, particularly the risk of uterine rupture recurrence, and managed her pregnancy progress carefully. We took great care to ensure that signs of a silent rupture were not missed on imaging examinations. A planned cesarean delivery was performed at 35 weeks of gestation, resulting in an uneventful pregnancy outcome. We report the details of our management of a subsequent pregnancy in a woman who had previously experienced uterine rupture with natural healing. Our findings may serve to support healthcare providers managing similar cases.

## Introduction

Uterine rupture is a significant pregnancy complication that can lead to catastrophic outcomes, such as massive bleeding and extensive blood transfusions, events that are closely associated with severe morbidity and mortality for both mother and fetus [1-4]. The primary treatment options include surgical interventions, such as hysterectomy or uterine preservation surgery [5,6]. When uterine preservation is successful, there is a concern for complications in future pregnancies, especially uterine rupture recurrence. According to a meta-analysis, the rate of uterine rupture recurrence is approximately 10% [7].

We previously documented a case of uterine rupture that healed spontaneously without surgical intervention [8]. The findings suggested that effective hemorrhage management may eliminate the necessity for surgery in such cases. However, the literature concerning subsequent pregnancies after spontaneous healing of uterine rupture remains limited.

In this report, we detail a case of subsequent pregnancy after uterine rupture with natural healing and outline our comprehensive management approach. Our aim is to support healthcare professionals in handling similar cases.

## Case presentation

**Figure 1:**
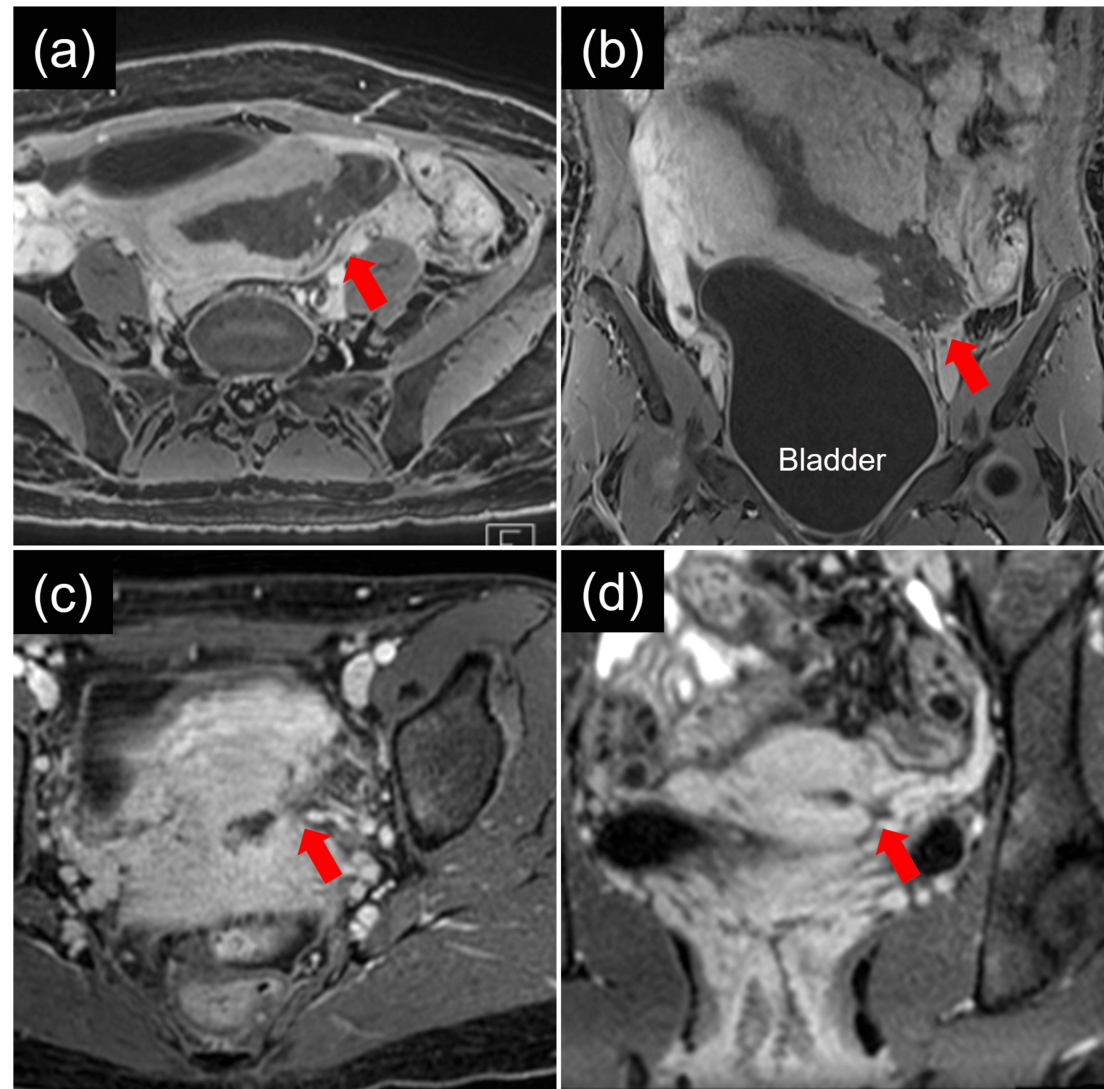
Magnetic resonance imaging of the previous uterine rupture. (a, b) Axial (a) and coronal (b) T1-weighted images immediately after the uterine rupture. A disruption in the myometrium is observed on the left side of the lower uterus. The broad ligament is preserved, leading to a diagnosis of incomplete uterine rupture. The red arrows indicate the uterine rupture site. These images have been revised from those published in our previous report. (c, d) Axial (c) and coronal (d) T1-weighted images at one year after the uterine rupture. Significant thinning of the myometrium is observed on the left side of the lower uterus, but the continuity is maintained. The red arrows indicate the uterine rupture scar.

A 27-year-old, gravida 2, para 1, woman was referred to our hospital at seven weeks of gestation for high-risk pregnancy management. She had previously experienced uterine rupture with postpartum hemorrhage [8]. Here, we briefly describe the previous situation. She was a 25-year-old primigravida with an uneventful pregnancy course. Her uterus was non-scarred. At 41 weeks of gestation, labor was induced using oxytocin, and vacuum-assisted delivery with fundal pressure was performed due to fetal bradycardia, resulting in a newborn weighing 3,274 grams. She experienced excessive postpartum hemorrhage, for which a uterine balloon was placed to achieve hemostasis. Ultrasound and magnetic resonance imaging (MRI) examinations revealed a discontinuity in the uterine myometrium, although the serosa remained intact. This led to a diagnosis of incomplete uterine rupture (Figures 1a, 1b). She recovered without the need for surgical intervention, and the rupture site healed spontaneously (Figures 1c, 1d). MRI revealed thinning on the left side of the lower uterus and a uterine rupture scar. However, these findings were not detected in an ultrasound examination. Menstruation resumed with no menstrual cycle abnormalities. Our follow-up concluded one year after delivery. Regarding future pregnancy, we discussed the potential for adverse perinatal outcomes with her and her partner. Should she become pregnant again, we advised her to visit us immediately.

At 15 months after the delivery, she conceived naturally. When she was first seen at seven weeks of gestation, transvaginal ultrasound revealed a single viable fetus that was growing appropriately. The uterine rupture scar was indistinct. We fully explained the risks of perinatal complications, especially rupture recurrence, to her and her partner. Beginning at 23 weeks of gestation, she was admitted to our facility for inpatient care to guarantee prompt intervention should a re-rupture occur. She was thoroughly educated about the early symptoms of uterine rupture, including abdominal pain, uterine contractions, and genital bleeding.

**Figure 2:**
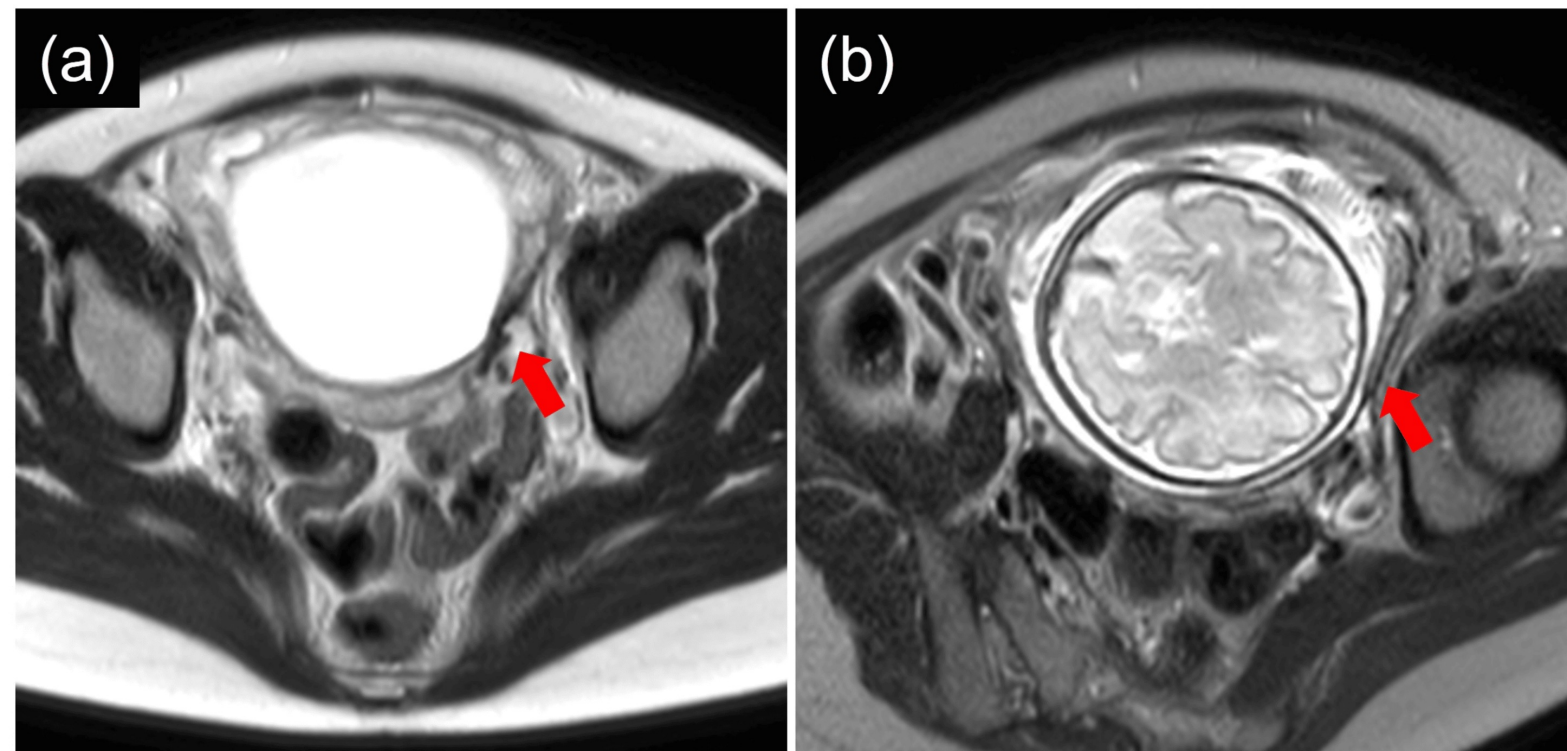
Magnetic resonance imaging during the current pregnancy. (a, b) Axial T2-weighted images at 24 (a) and 31 (b) weeks of gestation. Thinning of the myometrium is observed on the left side of the lower uterus. Although the normal uterine myometrium appears indistinct, no clear signs of a silent rupture are evident. The red arrows indicate the uterine rupture scar.

Ultrasound was performed weekly, and an MRI was conducted at 24 and 31 weeks of gestation to check for silent rupture. Localized thinning of the myometrium was observed on the lower left side of the uterus, but there were no signs of re-rupture (Figure 2). The locations of the placenta and scar were confirmed, leading to a detailed discussion regarding cesarean delivery. After weighing the risks of re-rupture against premature delivery, the obstetrics and neonatology teams discussed the matter and opted for planned cesarean delivery at 35 weeks and five days of gestation.

**Figure 3:**
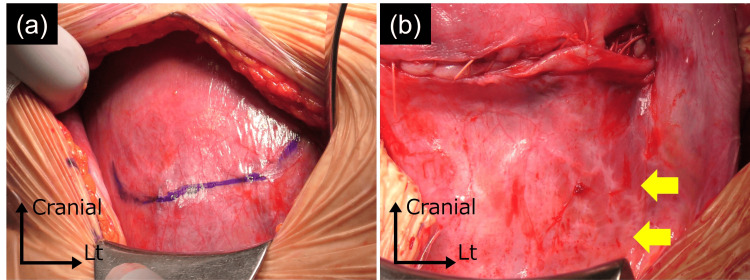
Findings at the time of surgery. (a) A line was drawn along the incision line, positioned slightly cephalad to the lower segment of the uterus. (b) After suturing the uterus, identification of the uterine rupture scar proved difficult. The yellow arrows indicate the presumed location of the uterine rupture scar.

The cesarean delivery was performed through a U-shaped incision, slightly cephalad to the lower uterine segment, to avoid the uterine rupture scar (Figure 3a). As a result, a healthy baby boy weighing 2,418 grams was delivered safely. The total intraoperative blood loss was 455 mL. During the surgery, identification of the uterine rupture scar was difficult (Figure 3b). The patient had an uneventful postoperative recovery and was discharged after six days. The newborn was admitted to the neonatal intensive care unit due to the preterm delivery and discharged on day 17.

## Discussion

Uterine rupture can heal spontaneously without the need for surgical intervention. Under these conditions, pregnancy and delivery can proceed without complications, as demonstrated by our case. Our meticulous management approach may serve as a valuable guideline for similar cases.

The incidence of uterine rupture has increased in parallel with increases in cesarean delivery and fibroid surgery, resulting in higher rates of rupture [9]. Uterine rupture can also occur in an unscarred uterus, designated atypical uterine rupture [3]. Our case represents an example of atypical uterine rupture, possibly related to the use of uterine fundal pressure [10]. Treatment for uterine rupture typically involves surgical interventions, such as hysterectomy or repair, although the choice of treatment can vary depending on study characteristics and regional practices [5,6,9,11]. In our case, surgical intervention was unnecessary because of the incomplete nature of the uterine rupture, which prevented continuous bleeding into the abdominal cavity. The bleeding was likely controlled by the application of continuous pressure to the bleeding site through the uterine balloon tamponade.

**Figure 4:**
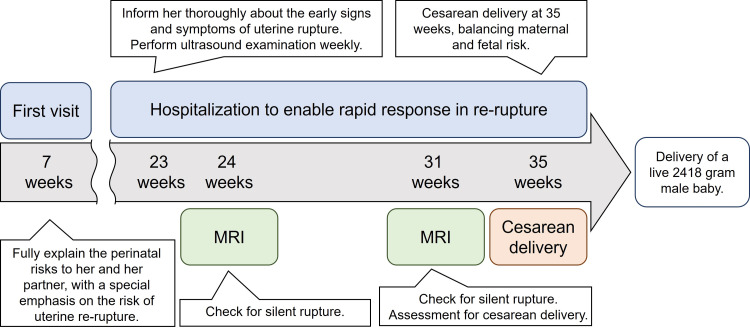
Timeline of events in the case.

As observed in our case, uterine rupture can heal spontaneously. However, the risk of uterine rupture recurrence in subsequent pregnancies in patients with a preserved uterus remains unknown. Herein, we present our management approach (Figure 4). Our management approach is not one-size-fits-all and can vary depending on the background characteristics and medical care regions of individual patients.

When encountering a case of uterine rupture that has healed naturally, it is crucial to provide information and education to the patient regarding a future pregnancy. This should include a discussion on the potential risk of rupture recurrence. In the event of a subsequent pregnancy, management at a center capable of offering multidisciplinary treatment is essential. Moreover, it is important for the patient to seek immediate medical attention if pregnancy is suspected. Meanwhile, it is necessary to assess the location and extent of the uterine rupture scar while the patient is not pregnant. Upon confirmation of pregnancy, it is vital to reiterate the perinatal risks to both the patient and their partner during the early stage of pregnancy.

According to a meta-analysis, the average time for uterine rupture recurrence in a subsequent pregnancy is 32.5 weeks of gestation (95% confidence interval, 29.9-35.1 weeks) [7]. Based on this 95% confidence interval, hospitalization for monitoring at approximately 28 weeks of gestation can be recommended. If hospitalization is not possible, potential relocation to a place with good medical access is advisable. This is because complications for both the mother and newborn after uterine rupture can be improved by rapid intervention, especially if the intervention occurs within 20 minutes [12,13]. In our case, the pregnancy followed a uterine rupture with natural healing and was deemed to be very high risk, and thus hospitalization from 23 weeks of gestation was initiated.

During the pregnancy, the patient was educated to immediately inform us if she experienced any early symptoms of uterine rupture, such as abdominal pain, uterine contractions, or genital bleeding. Ultrasound examinations were conducted to check the scarred area of the uterus for the possibility of silent rupture [14-16]. Since evaluation by ultrasound can sometimes be challenging, it is advisable to also use MRI [14]. The MRI examinations clarified the positions of both the uterine rupture scar and placenta and assisted during the sharing of surgical plans for cesarean delivery among the medical team.

Determining the optimal timing for cesarean delivery necessitates thorough consultations with obstetricians and neonatologists. In patients with a history of uterine rupture, cesarean delivery occurs at an average time of 35.8 weeks of gestation (95% confidence interval, 35.0-36.6 weeks) [7]. Frank et al. suggested that planned delivery should occur between 34 and 36 weeks of gestation. Given these insights, aiming for delivery within this timeframe is believed to strike a delicate balance between minimizing the risk of recurrence for the mother and reducing the risk of preterm complications for the newborn [17]. Nevertheless, the final decision should consider various factors, including the location of the previous uterine rupture and the gestational age at which the rupture happened. For pregnancy after a uterine rupture with natural healing, the possibility of earlier delivery may be considered. In our case, the previous rupture occurred at 41 weeks of gestation in the lower part of the uterus, and the current pregnancy was progressing without problems. Therefore, we chose to proceed with delivery at 35 weeks of gestation.

Regarding the method of delivery, we had to choose a planned cesarean delivery [18]. Before the surgery, it was necessary to have a detailed meeting with the anesthesiology department. We also confirmed the location of the uterine rupture scar and decided on the incision line for the cesarean delivery. In our case, the scar was located on the lower left side of the uterus, and thus we selected a uterine incision that would avoid the relevant area.

## Conclusions

This case demonstrates that successful management of a subsequent pregnancy following spontaneous uterine rupture with spontaneous healing is feasible without major complications. Early hospitalization, detailed patient education on uterine rupture signs, regular imaging, and a planned cesarean delivery were integral to achieving a favorable outcome for both mother and child. Although spontaneous healing after uterine rupture is rare, acknowledging this potential enables more personalized perinatal care. This case emphasizes the necessity of individualized management strategies and multidisciplinary collaboration in high-risk pregnancies, offering critical insights for optimizing maternal and fetal safety.

## References

[1] Ofir K, Sheiner E, Levy A, Katz M, Mazor M (2003). Uterine rupture: risk factors and pregnancy outcome. Am J Obstet Gynecol.

[2] Finnsdottir SK, Maghsoudlou P, Pepin K, Gu X, Carusi DA, Einarsson JI, Rassier SL (2023). Uterine rupture and factors associated with adverse outcomes. Arch Gynecol Obstet.

[3] Vandenberghe G, Vierin A, Bloemenkamp K (2023). Incidence and outcomes of uterine rupture in women with unscarred, preterm or prelabour uteri: data from the international network of obstetric survey systems. BJOG.

[4] Hofmeyr GJ, Say L, Gülmezoglu AM (2005). WHO systematic review of maternal mortality and morbidity: the prevalence of uterine rupture. BJOG.

[5] Thakur A, Heer MS, Thakur V, Heer GK, Narone JN, Narone RK (2001). Subtotal hysterectomy for uterine rupture. Int J Gynaecol Obstet.

[6] Alemayehu W, Ballard K, Wright J (2013). Primary repair of obstetric uterine rupture can be safely undertaken by non-specialist clinicians in rural Ethiopia: a case series of 386 women. BJOG.

[7] Sugai S, Yamawaki K, Haino K, Yoshihara K, Nishijima K (2023). Incidence of recurrent uterine rupture: a systematic review and meta-analysis. Obstet Gynecol.

[8] Sugai S, Haino K, Yamawaki K, Nishijima K, Enomoto T (2022). Spontaneous healing of uterine rupture causing postpartum hemorrhage. Eur J Obstet Gynecol Reprod Biol.

[9] Al-Zirqi I, Stray-Pedersen B, Forsén L, Daltveit AK, Vangen S (2016). Uterine rupture: trends over 40 years. BJOG.

[10] Hasegawa J, Sekizawa A, Arakaki T, Ikeda T, Ishiwata I, Kinoshita K (2020). Decline number of uterine fundal pressure maneuver in Japan recent 5 years. J Obstet Gynaecol Res.

[11] Chibber R, El-Saleh E, Al Fadhli R, Al Jassar W, Al Harmi J (2010). Uterine rupture and subsequent pregnancy outcome--how safe is it? A 25-year study. J Matern Fetal Neonatal Med.

[12] Leung AS, Leung EK, Paul RH (1993). Uterine rupture after previous cesarean delivery: maternal and fetal consequences. Am J Obstet Gynecol.

[13] Al-Zirqi I, Daltveit AK, Vangen S (2018). Infant outcome after complete uterine rupture. Am J Obstet Gynecol.

[14] Hruban L, Jouzova A, Janku P (2023). Conservative management of complete fetal expulsion into the abdominal cavity after silent uterine rupture - case report. BMC Pregnancy Childbirth.

[15] Fukutani R, Hasegawa J, Arakaki T, Oba T, Nakamura M, Sekizawa A (2017). Silent uterine rupture occluded by intestinal adhesions following laparoscopic myomectomy: a case report. J Obstet Gynaecol Res.

[16] Chen SH, Du XP (2019). Silent spontaneous posterior uterine rupture of a prior caesarean delivery at 36 weeks of gestation. BMC Pregnancy Childbirth.

[17] Frank ZC, Lee VR, Hersh AR, Pilliod RA, Caughey AB (2021). Timing of delivery in women with prior uterine rupture: a decision analysis. J Matern Fetal Neonatal Med.

[18] Dabi Y, Bouaziz J, Burke Y, Nicolas-Boluda A, Cordier AG, Chayo J, Cohen SB (2023). Outcome of subsequent pregnancies post uterine rupture in previous delivery: a case series, a review, and recommendations for appropriate management. Int J Gynaecol Obstet.

